# Regional Heterogeneity of Sensorimotor BOLD Lateralization During Unilateral Hand Movement

**DOI:** 10.21203/rs.3.rs-11254024/v1

**Published:** 2026-10-27

**Authors:** Xu Chang, Hongyu Zhou, Qingyun Wang, Zixiao Li, Yongjun Wang

**Affiliations:** Beijing Tiantan Hospital, Capital Medical University; Beijing Tiantan Hospital, Capital Medical University; Beihang University; Beijing Tiantan Hospital, Capital Medical University; Beijing Tiantan Hospital, Capital Medical University

**Keywords:** Sensorimotor cortex, hemispheric lateralisation, task-based fMRI, regional heterogeneity, motor execution, independent-cohort comparison

## Abstract

Unilateral hand movement engages contralateral sensorimotor cortex, but the balance of contralateral and ipsilateral responses can differ across regions. We quantified regional blood oxygen level-dependent lateralization in 20 CoSpine participants and compared corresponding endpoints in 100 unrelated Human Connectome Project participants. Bilateral Brainnetome parcels represented primary motor, primary somatosensory, dorsal premotor, and ventral premotor regions. Lateralization averaged the contralateral-minus-ipsilateral activation contrast across hands. CoSpine analyses used refitted activation models without physiological-recording regressors, following input qualification and correction of anatomical-mask mapping. Analyses retained five within-participant regional contrasts and a covariance-aware bootstrap sensitivity test. CoSpine composite lateralization averaged 28.059 contrast-of-parameter-estimate units (95% confidence interval 23.718–32.551). The covariance-aware analysis supported regional heterogeneity (p < 0.001). Primary motor and somatosensory regions had the largest mean lateralization, dorsal premotor cortex an intermediate mean, and ventral premotor cortex a mean near zero. Four regional contrasts survived Holm correction; the primary motor–somatosensory difference was 3.653 units (95% confidence interval - 4.559 to 13.112; adjusted p = 0.462). The independent cohort showed a similar regional pattern, with a supported primary motor–somatosensory difference and weak positive ventral premotor lateralization. These results characterize regional variation in hemispheric response balance beyond the general observation of contralateral activation. The shared atlas-level pattern across distinct motor paradigms supports a differentiated account of sensorimotor lateralization.

## 1 Introduction

Unilateral hand movement provides a well-established model for investigating hemispheric organization in the human sensorimotor system. Movement execution engages precentral and postcentral cortex, with the precentral hand knob providing an anatomical landmark for the primary motor hand area (Porro et al., 1996; Yousry et al., 1997). Although contralateral responses predominate, the strength of this preference can vary across the regions involved in the same movement. Characterizing this variation is essential for understanding how hemispheric specialization is distributed across primary motor, somatosensory, and premotor cortex. A regional analysis can reveal differences that are obscured when a single overall measure summarizes sensorimotor lateralization.

Activation and lateralization describe complementary aspects of task-related brain responses. Activation measures the response relative to baseline, whereas hand-aligned lateralization compares homologous regions contralateral and ipsilateral to the moving hand. Both hemispheres therefore contribute to the lateralization estimate. Functional magnetic resonance imaging (fMRI) studies show that ipsilateral motor recruitment varies with the complexity of unimanual actions (Verstynen et al., 2005). Multivoxel analyses further demonstrate that ipsilateral cortex can represent individual fingers even when its mean blood-oxygen-level-dependent (BOLD) response is suppressed (Diedrichsen et al., 2013). These findings motivate examining lateralization as a measure of hemispheric response balance alongside regional activation.

Primary motor cortex (M1), primary somatosensory cortex (S1), dorsal premotor cortex (PMd), and ventral premotor cortex (PMv) provide a focused anatomical framework for this comparison. These regions span motor execution, the sensory consequences of movement, and premotor processing, and each has a corresponding territory in the opposite hemisphere. Primate recordings have identified distinct dorsal and ventral premotor contributions to action planning (Hoshi & Tanji, 2006), while human imaging has revealed effector-specific motor territories interspersed with regions involved in broader action organization (Gordon et al., 2023). This functional diversity motivates testing whether contralateral preference differs across sensorimotor regions. Bilateral parcels from the Brainnetome atlas provide consistent anatomical definitions independent of observed task responses (Fan et al., 2016).

Examining corresponding regions in independent cohorts can establish which features of lateralization recur across experimental settings. The CoSpine dataset includes auditory-paced hand grasping performed in separate left- and right-hand runs acquired with a simultaneous cortico-spinal fMRI protocol (Wei et al., 2025). The Human Connectome Project (HCP) provides visually cued finger-movement tasks, with both hands and other effectors represented within each motor run, together with standardized acquisition and preprocessing procedures (Barch et al., 2013; Glasser et al., 2013; Van Essen et al., 2013). Applying the same anatomical definitions and lateralization measure to both datasets enables comparison of regional patterns across these distinct motor paradigms.

This study aimed to characterize regional variation in hand-aligned BOLD lateralization in CoSpine and examine whether a comparable pattern was present in an independent HCP cohort. We quantified lateralization as the contralateral-minus-ipsilateral activation contrast averaged across hands. An equally weighted four-region composite summarized the overall hemispheric bias, while regional estimates and five within-participant contrasts characterized its distribution across M1, S1, PMd, and PMv. Covariance-aware sensitivity analyses additionally assessed overall regional heterogeneity. Together, these analyses examined the regional organization of hemispheric preference and its consistency across cohorts.

## 2 Methods

### 2.1 Participants and datasets

We analyzed motor-task functional magnetic resonance imaging (fMRI) from the CoSpine motor dataset (OpenNeuro ds005884; Wei et al., 2025) and the Human Connectome Project (HCP) 100 Unrelated Subjects sample (Van Essen et al., 2013). CoSpine provided the regional activation analysis, and HCP provided an independent cohort in which we examined corresponding regional measures. Figure 1 summarises the study design.

The CoSpine motor dataset comprised 22 right-handed healthy adults. Inclusion required both hand-task runs, consistent imaging and task records, successful first-level modeling, and adequate bilateral coverage of all regions used in a given analysis. Two participants were excluded because acquisition-to-task correspondence remained unresolved for one or both runs. The final sample included 20 participants and 40 runs; mean age was 52.55 years (SD 9.96; range 37–74), with 10 male and 10 female participants. All 20 met the four-region coverage criterion. The HCP sample included 100 unrelated participants with two motor runs each, all with complete four-region data. Demographic summaries for these exact 100 participants were unavailable in the retained records. Table 1 shows sample characteristics; Supplementary Material 1 (Tables S1–S2) details participant disposition and regional coverage.

This secondary analysis involved no new participant recruitment or data collection. The Ethics Committee of the Institute of Psychology, Chinese Academy of Sciences approved the original CoSpine study (Approval No. H20055), and all participants provided written informed consent (Wei et al., 2025). The HCP data were collected as part of the original project under its ethics approvals and informed-consent procedures (Van Essen et al., 2013).

### 2.2 Motor tasks and MRI acquisition

CoSpine participants performed auditory-paced grasping with a 6.5-cm dynamometer in separate left-hand and right-hand runs. Each run comprised 12 movement blocks, each containing eight grasps at 1 Hz over 8 s, separated by jittered rest (Wei et al., 2025). Participant-specific event files supplied movement onsets and durations. In the original CoSpine study, images were acquired on a 3-T Siemens Prisma scanner with a 64-channel head–neck coil (Wei et al., 2025). Functional echo-planar imaging (EPI) used a repetition time (TR) of 2.68 s, echo time (TE) of 27 ms, flip angle of 80°, 70 contiguous slices, and 1.5 × 1.5 × 4-mm voxels, with in-plane and simultaneous-multislice acceleration factors of 2. T1-weighted MPRAGE images used TR/TE/inversion time of 2300/2.28/900 ms, a flip angle of 8°, and 1-mm isotropic resolution. Thirty-nine analyzed runs contained 115 saved volumes, and one contained 170. All saved volumes were retained; event times were used without additional onset shifts.

The HCP motor task used visual cues to prompt movements of the left or right fingers, left or right toes, or tongue (Barch et al., 2013). Each run contained two 12-s blocks per movement type, preceded by 3-s cues, and three 15-s fixation blocks. In the HCP, images were acquired on a customized 3-T Siemens Skyra scanner with a 32-channel head coil (Van Essen et al., 2013). Gradient-echo EPI used TR = 720 ms, TE = 33.1 ms, a 52° flip angle, a multiband factor of 8, a 104 × 90 matrix, and 72 slices at 2-mm isotropic resolution. Each participant contributed two 284-volume runs, acquired with left-to-right (LR) and right-to-left (RL) phase encoding. Each run included both hand conditions; LR and RL refer to phase encoding, not the moving hand (Van Essen et al., 2013).

### 2.3 Image processing and activation models

CoSpine images were processed using the FMRIB Software Library (FSL; Jenkinson et al., 2012). We applied susceptibility-distortion correction using reversed-phase-encoding images, followed by slice-timing correction, rigid-body motion correction, EPI-to-T1 registration, and transformation to MNI152 2-mm space. We applied no additional spatial smoothing or regression of recorded physiological signals. This processing branch is referred to as non-PNM, where PNM denotes physiological-noise modeling. HCP analyses used the official S1200 minimally preprocessed volumetric images, which underwent gradient-distortion correction, motion and susceptibility-distortion correction, anatomical registration, and transformation to MNI space (Glasser et al., 2013). No additional smoothing was applied to HCP images. Stage-specific processing details and available software-version records are provided in Supplementary Material 1, Section S3.

For CoSpine, the analysis mask combined anatomical brain coverage and valid functional sampling. We transformed the T1 brain mask to native EPI space using inverse EPI-to-T1 registration and nearest-neighbor interpolation, intersected it with voxels containing finite, nonzero signal in at least 95% of time points, and transformed it to MNI space with nearest-neighbor interpolation. Intersection with the standard MNI brain mask defined the final model mask. All 40 activation models were fitted with this mask definition. Quality control assessed anatomical alignment, design rank, residual degrees of freedom, contrast specification, finite estimates, and model completion. The mask correction and matched-processing sensitivity analysis are documented in Supplementary Material 1, Sections S3 and S5.

First-level general linear models were fitted separately for each run using FSL 6.0.7.22 and the fMRI Expert Analysis Tool (FEAT). CoSpine movement blocks were modeled relative to implicit rest using a double-gamma hemodynamic response function (HRF) and its temporal derivative. Nuisance regressors included six rigid-body motion parameters, their six backward first differences, and one impulse regressor for each volume with Power framewise displacement (FD) > 0.5 mm (Power et al. 2012). High-motion volumes were retained. Models used 100-s high-pass filtering, grand-median intensity scaling to 10,000, and FILM prewhitening. The movement contrast assigned weight 1 to the canonical movement coefficient and 0 to the derivative and nuisance columns.

HCP LR and RL runs were modeled separately using FSL 6.0.7.22. Six regressors represented the five movement conditions and visual cues, each convolved with the double-gamma HRF and accompanied by a temporal derivative. Models included the supplied 12 motion and motion-derivative columns, run baseline modeling, 100-s high-pass filtering, and FILM prewhitening. FD was calculated from backward differences of the first six motion columns, with translations in millimetres, rotations converted from degrees to radians and a 50-mm head radius; the initial difference was zero. Each volume with FD > 0.5 mm received an impulse regressor, without neighboring-volume expansion or volume deletion. Left-hand and right-hand movement contrasts were estimated relative to implicit fixation, with the other movement conditions and cues included in the model. These were locally fitted volumetric models, not the released cross-run CIFTI contrast maps.

We measured regional activation from unthresholded contrast-of-parameter-estimate (COPE) images. COPE values retain the scaled-signal units of the fitted model and were not interpreted as percentage signal change.

### 2.4 Regions of interest and activation lateralization

Four bilateral regions of interest (ROIs) were defined using the 246-region Brainnetome atlas (Fan et al., 2016): primary motor cortex (M1; A4ul, labels 57/58), primary somatosensory cortex (S1; A1/2/3ulhf, 155/156), dorsal premotor cortex (PMd; A6cdl, 55/56) and ventral premotor cortex (PMv; A6cvl, 63/64). The S1 parcel includes upper-limb, head, and face territory. We used the same anatomical definitions in both cohorts. Each unilateral parcel was intersected with the run-specific analysis mask and required at least 80% coverage, calculated as the retained voxel count divided by the full parcel voxel count on the same grid. We checked atlas, mask, and COPE dimensions and affines for correspondence; we did not resample the atlas during extraction.

For each hand condition and hemisphere, regional activation was the unweighted mean COPE across all voxels in the effective ROI, without activation-threshold selection. Regional lateralization was calculated as the contralateral-minus-ipsilateral activation difference, averaged across the two hands:

$$D_{r}=\frac{\left(C_{r,RH,L}-C_{r,RH,R}\right)+\left(C_{r,LH,R}-C_{r,LH,L}\right)}{2}$$

Here, C denotes the mean movement COPE in region r; RH and LH denote the right- and left-hand tasks, and L and R denote anatomical hemispheres. Positive values indicate greater contralateral activation. Both hands and both hemispheres were required, and missing values were not imputed. An equally weighted four-region average summarised overall sensorimotor lateralization:

$$D_{motor}=\frac{D_{M1}+D_{S1}+D_{PMd}+D_{PMv}}{4}$$

For HCP, regional lateralization was calculated within each LR and RL run and then averaged equally across runs within participants. The four-region average and paired regional comparisons used complete within-participant observations. These measures are signed activation differences, not normalized laterality ratios. CoSpine and HCP estimates were analyzed on their respective COPE scales without pooling raw values across cohorts.

### 2.5 Statistical analysis and sensitivity analyses

Participants were the independent statistical units. We calculated group means from participant-level lateralization values and derived pointwise 95% confidence intervals from percentile limits from 10,000 participant bootstrap resamples. Standardized effects were calculated as the mean divided by the sample standard deviation (Cohen’s dz). In CoSpine, we tested the four-region average and individual regional values against zero using exact two-sided sign-flip tests based on the absolute sample mean. All 2^n^ participant-level sign assignments were enumerated, including equally extreme values. This procedure assumes sign invariance under the null, commonly justified by symmetric participant-level errors (Winkler et al., 2014). The four-region average was tested separately; the four regional tests formed one Holm correction family (Holm, 1979).

Five previously specified within-participant regional contrasts were evaluated: M1 - PMv, S1 - PMv, PMd - PMv, mean(M1, S1) - mean(PMd, PMv), and M1 - S1. CoSpine contrasts used exact two-sided mean sign-flip tests and 10,000-resample percentile confidence intervals. Holm correction was applied across all five comparisons. The same contrast definitions and five-comparison family were used in HCP, with 100,000 Monte Carlo mean sign flips and 10,000 participant bootstrap resamples for intervals.

HCP tests of the four-region average and each region against zero used exact two-sided binomial sign tests, with a null positive-value probability of 0.5 among nonzero observations. The four regional tests formed a separate Holm family. These tests assess sign probability, whereas the CoSpine sign-flip tests use the mean; we retained the original cohort-specific tests. All tests were two-sided, with p < 0.05 or Holm-adjusted p < 0.05, as applicable. Confidence intervals were not multiplicity-adjusted.

We assessed regional heterogeneity in each cohort using a covariance-aware Wald-type statistic (WTS) as a revision-stage sensitivity analysis (Bathke et al., 2018; Friedrich et al., 2019). The null hypothesis was that the four regional means were equal. A rank-three contrast compared M1, S1, and PMd with PMv, using the full within-participant covariance matrix. The null distribution was approximated with 99,999 zero-mean multivariate-normal bootstrap samples generated with the observed covariance; covariance and WTS were re-estimated in each sample. The method accommodates unequal regional variances and within-participant dependence; its asymptotic justification assumes independent participant vectors, finite fourth moments, and nonsingular contrast covariance. Monte Carlo and parametric-bootstrap p-values were calculated as (b + 1)/(B + 1), where b counts statistics at least as extreme as observed, and B is the number of resamples. All five regional contrasts were reported independently of the WTS result.

We assessed processing sensitivity by comparing activation models before and after correcting anatomical-mask mapping within the same eligible participants. We tested paired changes in the four-region average and four regional measures using exact sign flips, 10,000-resample percentile intervals, and an additional five-test Holm family. Changes in sample composition were described separately from these within-participant processing differences. Run-order sensitivity used a regression of the four-region average on observed order (right-hand-first = 0; left-hand-first = 1), with heteroskedasticity-consistent HC3 covariance. Regional profile differences by order were evaluated using a repeated-measures interaction statistic and 100,000 participant-level order-label permutations, retaining each participant’s four-region vector. Run order was observed, not randomized. Specifications and full results are provided in Supplementary Material 1, Sections S5–S6.

The current regional activation analysis was established during revision. We recorded eligibility and processing rules before examining the new group results, after historical findings were known; we retained the regional measures and five contrast definitions. WTS, processing, and run-order analyses remain revision-stage sensitivities. The analysis history and treatment of earlier results are documented in Supplementary Material 1, Section S1. Regional extraction and statistics used Python, with software versions and implementation details in Supplementary Material 1, Sections S3–S4. Numerical results are supplied in Supplementary Material 2.

## 3 Results

### 3.1 Participant characteristics and regional coverage

The final CoSpine sample comprised 20 right-handed participants contributing 40 hand-task runs. Mean age was 52.55 years (SD 9.96; range 37–74), and 10 participants were female. Two of the original 22 participants were excluded because acquisition-to-task correspondence remained unresolved for one or both hand runs. All included participants met the 80% bilateral coverage criterion for M1, S1, PMd, and PMv, providing complete observations for the four-region average and all regional comparisons.

The HCP sample comprised 100 unrelated participants, each contributing LR and RL motor runs, for a total of 200 runs. All participants had complete data and adequate coverage for the four bilateral regions. Table 1 summarises sample characteristics, with participant disposition and regional coverage reported in Supplementary Material 1 (Tables S1–S2 and Fig. S1).

### 3.2 Regional activation lateralization in CoSpine

Mean lateralization across the four regions was positive in all 20 participants, averaging 28.059 COPE units (95% CI 23.718–32.551; Cohen’s dz = 2.721; exact sign-flip p < 0.001). Regional means differed in the covariance-aware sensitivity analysis (WTS = 246.378; bootstrap p < 0.001).

M1 and S1 showed the largest mean contralateral activation differences, at 52.686 COPE units (95% CI 43.062–63.805) and 49.033 COPE units (95% CI 43.057–55.255), respectively. PMd showed a smaller positive mean of 10.661 COPE units (95% CI 7.420–14.403). All three regions remained significant after correction across the four regional tests (Holm-adjusted p < 0.001). Mean PMv lateralization was close to zero (− 0.144 COPE units; 95% CI − 2.901 to 2.406; adjusted p = 0.917), with positive differences in 12 participants and negative differences in eight (Fig. 2A, C; Table 2).

Four of the five regional contrasts survived Holm correction: M1 - PMv, S1 - PMv, PMd - PMv, and mean(M1, S1)-mean(PMd, PMv) (all adjusted p < 0.001). The M1 - S1 difference was 3.653 COPE units (95% CI - 4.559 to 13.112; adjusted p = 0.462). Participant-level patterns and group estimates are shown in Fig. 3A, C, E and Table 3.

### 3.3 Regional lateralization in the independent HCP cohort

The HCP cohort showed a similar broad regional pattern. Mean lateralization across the four regions was positive in all 100 participants, averaging 35.947 HCP COPE units (95% CI 33.743–38.185; exact binomial sign-test p < 0.001). Covariance-aware sensitivity analysis supported differences among the regional means (WTS = 1072.557; bootstrap p < 0.001).

Mean lateralization was largest in M1 (69.470 COPE units; 95% CI 65.018–73.904) and S1 (53.907; 95% CI 50.593–57.308), followed by PMd (17.352; 95% CI 15.461–19.255). PMv showed a small positive mean of 3.061 COPE units (95% CI 0.643–5.454), with positive values in 61 participants and negative values in 39. All four regional sign tests survived Holm correction, including PMv (adjusted p = 0.035; Fig. 2B, D; Table 2).

All five regional contrasts survived Holm correction (adjusted p < 0.001). The M1 - S1 difference was 15.563 HCP COPE units (95% CI 11.706–19.381; Fig. 3B, D, F; Table 3). Thus, both cohorts showed prominent M1/S1 lateralization and the smallest mean difference in PMv, while HCP showed statistical support for M1 - S1 and positive PMv lateralization, but CoSpine did not. These are within-cohort findings rather than a direct test of between-cohort differences. Participant-level LR/RL comparisons are provided in Supplementary Material 1, Fig. S3.

### 3.4 Sensitivity analyses

We evaluated processing sensitivity within participants with valid measurements under both the previous and corrected anatomical-mask implementations. Among the 17 participants with complete four-region data under both implementations, mean lateralization across regions was 28.284 and 28.814 COPE units, respectively. The paired increase was 0.530 COPE units (Holm-adjusted p = 0.394). None of the five paired processing-change tests survived correction. The largest regional mean change occurred in M1 (+ 2.034 COPE units; n = 19; adjusted p = 0.236). These matched-sample comparisons were analyzed separately from changes in sample composition. Full estimates and confidence intervals are reported in Supplementary Material 1, Tables S5–S7 and Fig. S2.

Twelve CoSpine participants performed the left-hand run first and eight performed the right-hand run first. The estimated association between run order and four-region average lateralization was 4.322 COPE units for left-hand-first relative to right-hand-first (HC3 95% CI - 4.776 to 13.421; p = 0.352). The regional-profile-by-order interaction was also not significant (F = 0.055; permutation p = 0.972; Supplementary Material 1, Table S8).

## 4 Discussion

Hand-aligned BOLD lateralization varied across the four sensorimotor regions, with a similar broad pattern in CoSpine and the independent HCP cohort. M1 and S1 showed the largest mean contralateral preference, PMd an intermediate mean, and PMv the weakest preference. Within-participant comparisons supported greater average lateralization in the primary regions than in the premotor regions and distinguished PMd from PMv. Covariance-aware sensitivity analyses further supported regional heterogeneity in both cohorts. Together, these findings characterize how hemispheric response balance is distributed across sensorimotor cortex during unilateral hand movement.

Regional lateralization complements information on task activation magnitude. Because the measure incorporates both contralateral and ipsilateral responses, a region can show substantial task engagement while exhibiting only a small hemispheric difference. Research on manual preference shows that both contralateral activation and ipsilateral deactivation contribute to motor asymmetry (Tzourio-Mazoyer et al., 2021), while recent work across multiple tasks has examined distinct relationships between BOLD amplitude, hemispheric asymmetry, and performance (Tomasi & Volkow, 2025). In the present study, averaging the contralateral-minus-ipsilateral contrast across hands provided a consistent endpoint for comparing homologous cortical territories. The four-region composite summarized the overall contralateral bias, whereas regional estimates revealed the spatial variation underlying that summary.

The pronounced lateralization of M1 and S1 is consistent with the motor and sensory processes accompanying hand movement. Movement execution recruits precentral and postcentral cortex, and the precentral hand area provides an anatomical reference for motor activation (Porro et al., 1996; Yousry et al., 1997). Grasping and finger movement combine motor output with sensory feedback, providing a functional context for the strong contralateral preference observed in both primary regions. Their relative separation was less consistent across cohorts: HCP supported greater lateralization in M1 than in S1, whereas the CoSpine confidence interval encompassed differences in either direction. The shared finding is therefore prominent lateralization in both primary regions. The mean ordering provides a descriptive comparison, with the within-participant tests specifying which differences were supported.

Premotor regions exhibited a different hemispheric balance. PMd retained a positive contralateral preference in both cohorts, whereas PMv lateralization was close to zero in CoSpine and weakly positive in HCP. The supported PMd–PMv contrast identifies a dorsal–ventral distinction within the premotor cortex. This pattern is consistent with evidence that ipsilateral premotor responses contribute to unilateral movement: Horenstein et al. (2009) observed ipsilateral activation predominantly in PMd and PMv during complex finger tapping. Primate recordings also demonstrate distinct dorsal and ventral premotor contributions to action planning (Hoshi & Tanji, 2006), while human imaging reveals effector-specific territories interspersed with regions involved in broader action organization (Gordon et al., 2023). These findings provide a functional context for the regional differences observed here, although the present BOLD contrasts do not isolate the computations responsible for those differences.

Weak PMv lateralization warrants interpretation in terms of hemispheric balance. Ipsilateral motor recruitment varies with task complexity (Verstynen et al., 2005), and finger-specific information can be represented in ipsilateral cortex even when its mean BOLD response is suppressed (Diedrichsen et al., 2013). Thus, a small regional contrast does not imply limited involvement in movement. The near-zero CoSpine estimate is compatible with similar average responses across hemispheres, but establishing bilateral activation would additionally require examining each hemisphere’s response relative to baseline. The present result identifies PMv as the region with the weakest net contralateral preference among the four parcels examined.

The independent-cohort comparison extends this regional description across distinct motor paradigms. CoSpine used auditory-paced grasping in separate hand runs, whereas HCP used visually cued finger movements with both hands and other effectors represented within each run. The recurrence of prominent M1/S1 lateralization and weak PMv lateralization suggests that the broad pattern extends beyond a single task and acquisition setting. Hand dominance and movement demands can influence cortical responses, as shown in studies of graded grip force and manual preference (Alahmadi et al., 2015; Tzourio-Mazoyer et al., 2021). These factors, along with sampling and measurement precision, may contribute to finer differences in M1–S1 separation and PMv lateralization. Because these factors were not experimentally matched, the cross-cohort evidence concerns concordance of the regional pattern; differences between cohorts would require direct tests using comparable measurements.

The shared anatomical framework strengthens this comparison. Brainnetome parcels were defined independently of the observed task responses and provided corresponding bilateral territories in both datasets (Fan et al., 2016). Coverage checks and consistent extraction procedures supported comparisons within this framework. Nevertheless, the spatial scale of the conclusions remains that of the selected parcels. In particular, the S1 parcel includes upper-limb, head, and face territory, and the premotor parcels may encompass finer functional subdivisions. Individual functional localization would help determine how the observed regional pattern relates to hand-selective territories and variation in cortical organization across participants.

Processing sensitivity analyses addressed the effects of correcting anatomical-mask mapping. Within matched participants, none of the five changes in composite or regional lateralization remained significant after Holm correction. These comparisons were reported separately from changes in sample eligibility, allowing processing effects to be distinguished from sample-composition effects. They quantify the consequences of the correction, although their confidence intervals do not establish equivalence between implementations. The final interpretation rests on the corrected activation models and their associated regional estimates.

Several limitations guide the interpretation of these findings. The CoSpine sample of 20 participants limits precision, particularly for smaller regional differences. Motion regressors and frame-specific impulse regressors were included, but recorded cardiac and respiratory regressors were unavailable to the analyzed models, leaving potential residual physiological influences. Separate hand runs in CoSpine also permit run-specific effects to contribute to the hand comparison. The order analyses detected no association, but the non-random allocation and broad confidence interval limit the strength of that assessment. Inference additionally depends on the assumptions of the selected tests, including sign invariance for sign-flip testing, and the covariance-aware bootstrap analysis was a revision-stage sensitivity analysis. Across cohorts, differences in task design, acquisition, COPE scaling, and region-versus-zero tests constrain quantitative comparison; demographic summaries for the exact HCP subset were also unavailable.

The findings support a regionally differentiated account of sensorimotor lateralization, with prominent contralateral preference in primary motor and somatosensory cortex and a distinct dorsal–ventral premotor pattern. Reporting regional estimates alongside the overall composite makes this organization visible and provides a clearer basis for interpreting hemispheric differences during movement. Future studies combining matched motor tasks, counterbalanced hand conditions, physiological recordings, and individual functional localization could test the finer regional distinctions and identify the factors that shape them.

## 5 Conclusion

Hand-aligned sensorimotor lateralization differed across the specified Brainnetome regions, with prominent M1/S1 contrasts, smaller PMd contrasts, and the weakest PMv contrast. An independent HCP cohort showed a similar broad regional pattern, alongside finer differences in M1 - S1 and PMv. These findings identify atlas-level variation in hemispheric response balance as a useful complement to the general contralateral organization of unilateral hand movement.

## Supplementary Material

This is a list of supplementary files associated with this preprint. Click to download.

- MainTables13.docx
- SupplementaryMaterial1.pdf
- SupplementaryMaterial2.zip

Supplementary Material 1 (Supplementary_Material_1.pdf): supplementary methods, analysis-scope revision, participant disposition, coverage, full regional results, processing and run-order sensitivities, and supplementary figures.

Supplementary Material 2 (Supplementary_Material_2.zip): machine-readable regional results, CoSpine participant measures and eligibility/coverage records, processing-comparison tables, and data documentation. HCP participant-level redistribution is excluded from this public-facing package.

## Figures and Tables

**Figure 1:**
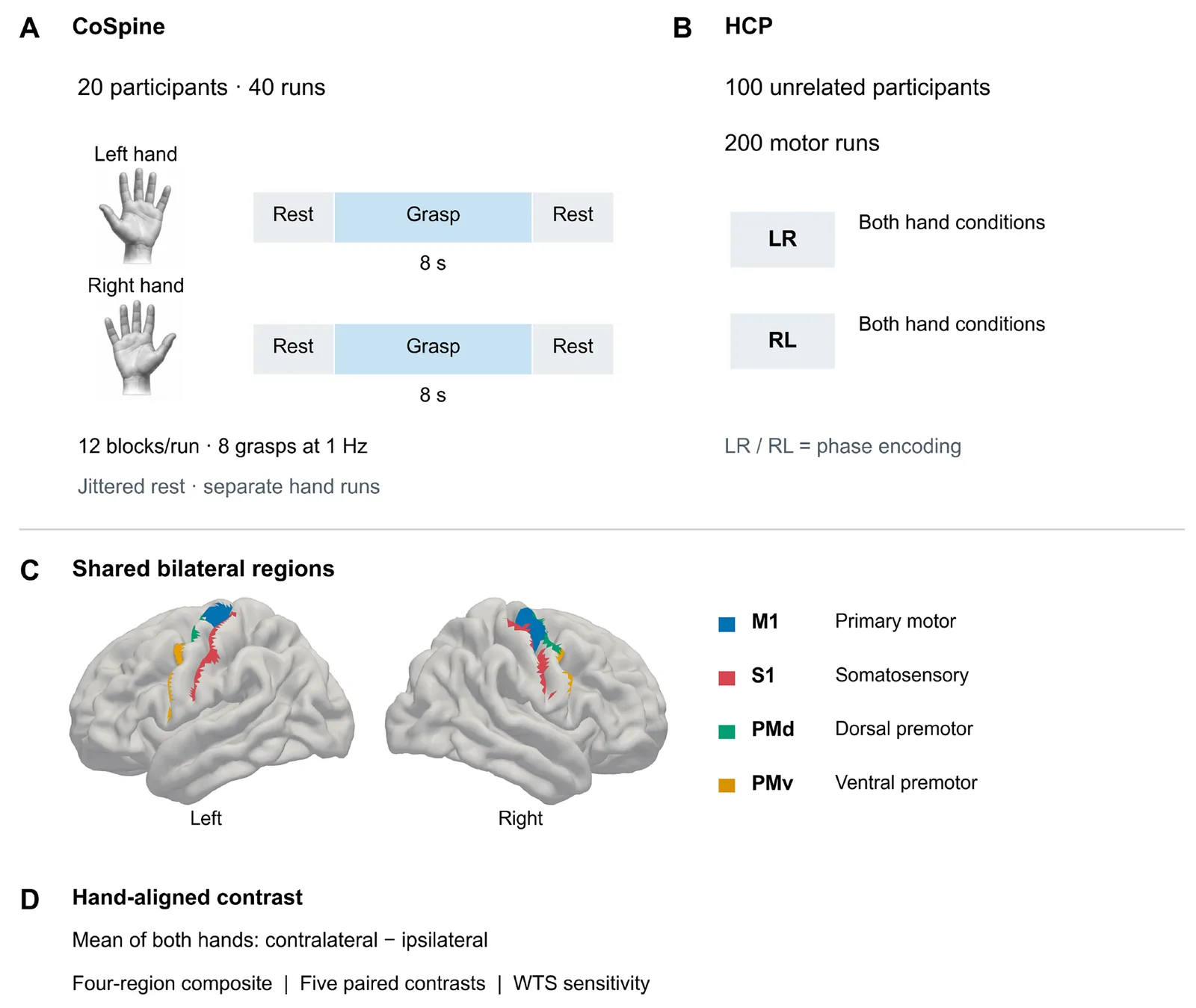
Study design and regional lateralization analysis. (A) CoSpine included 20 participants with separate left- and right-hand grasping runs (40 runs). Each run comprised 12 movement blocks, each containing eight grasps at 1 Hz, separated by jittered rest. The illustrated sequence is schematic. (B) HCP included 100 unrelated participants with two motor runs each; LR and RL indicate phase-encoding directions, and both hand conditions occurred within each run. (C) Bilateral Brainnetome regions displayed on a reference cortical surface: primary motor (M1), primary somatosensory (S1), dorsal premotor (PMd), and ventral premotor (PMv) cortex. Colors identify atlas parcels. (D) Regional lateralization averaged the contralateral-minus-ipsilateral movement response across hands, supplying the four-region composite, five paired regional comparisons, and Wald-type statistic (WTS) sensitivity analysis.

**Figure 2:**
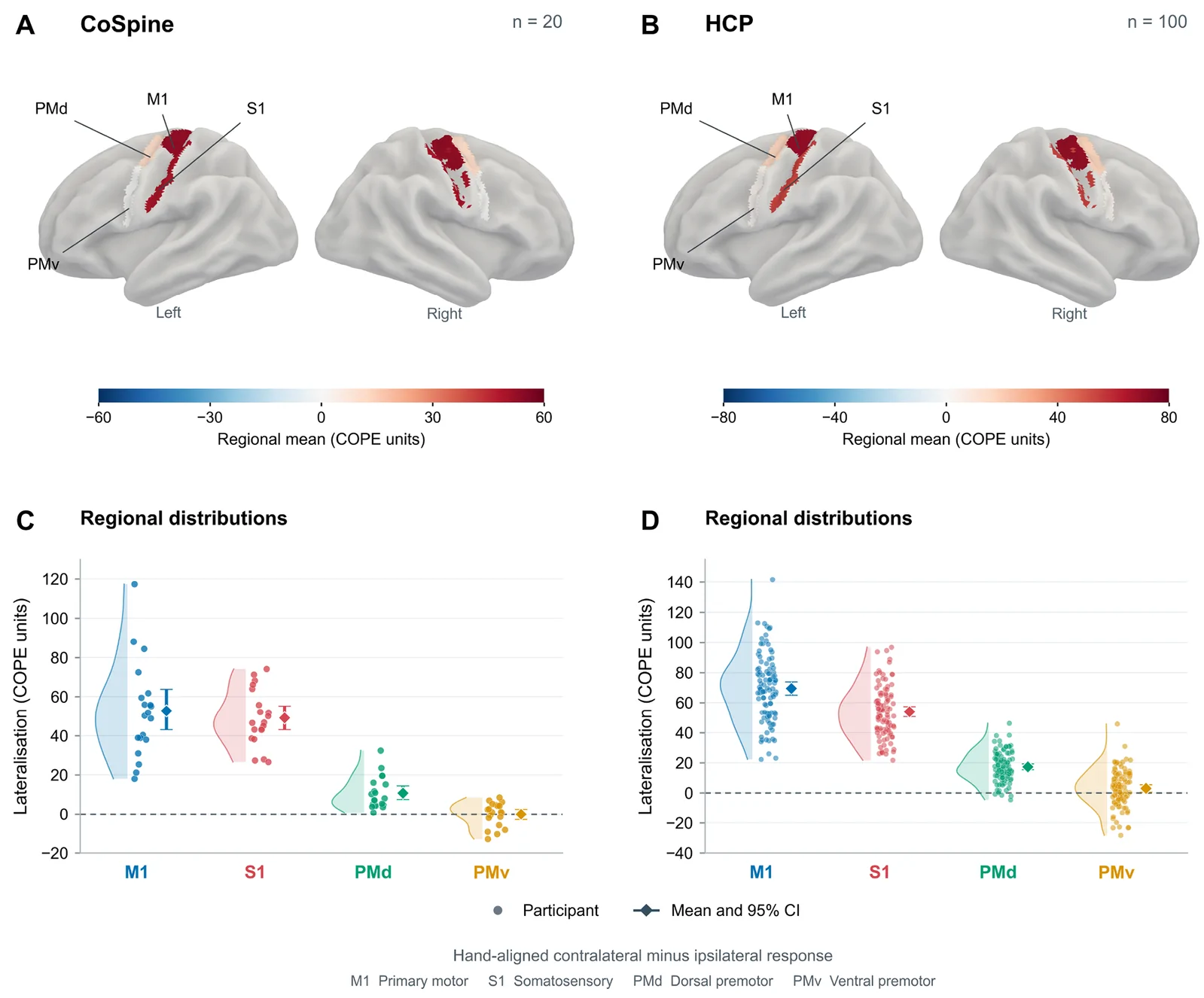
Regional distribution of activation lateralization. (A, B) Mean regional lateralization in CoSpine (n = 20) and HCP (n = 100), mapped onto bilateral Brainnetome parcels on a partially inflated reference cortical surface. Each hand-aligned regional mean is displayed on both hemispheres; colors represent regional contrasts, not hemisphere-specific activation or vertex-wise significance. Unselected cortex is grey. (C, D) Corresponding participant-level distributions. Half-violins show descriptive kernel densities restricted to the observed range and scaled to equal peak width; dots represent participants. Diamonds and error bars indicate means and pointwise 95% percentile-bootstrap confidence intervals from 10,000 participant resamples. Dashed lines denote zero. Color scales and axes retain cohort-specific contrast-of-parameter-estimate (COPE) units. M1, primary motor; S1, primary somatosensory; PMd, dorsal premotor; PMv, ventral premotor cortex.

**Figure 3:**
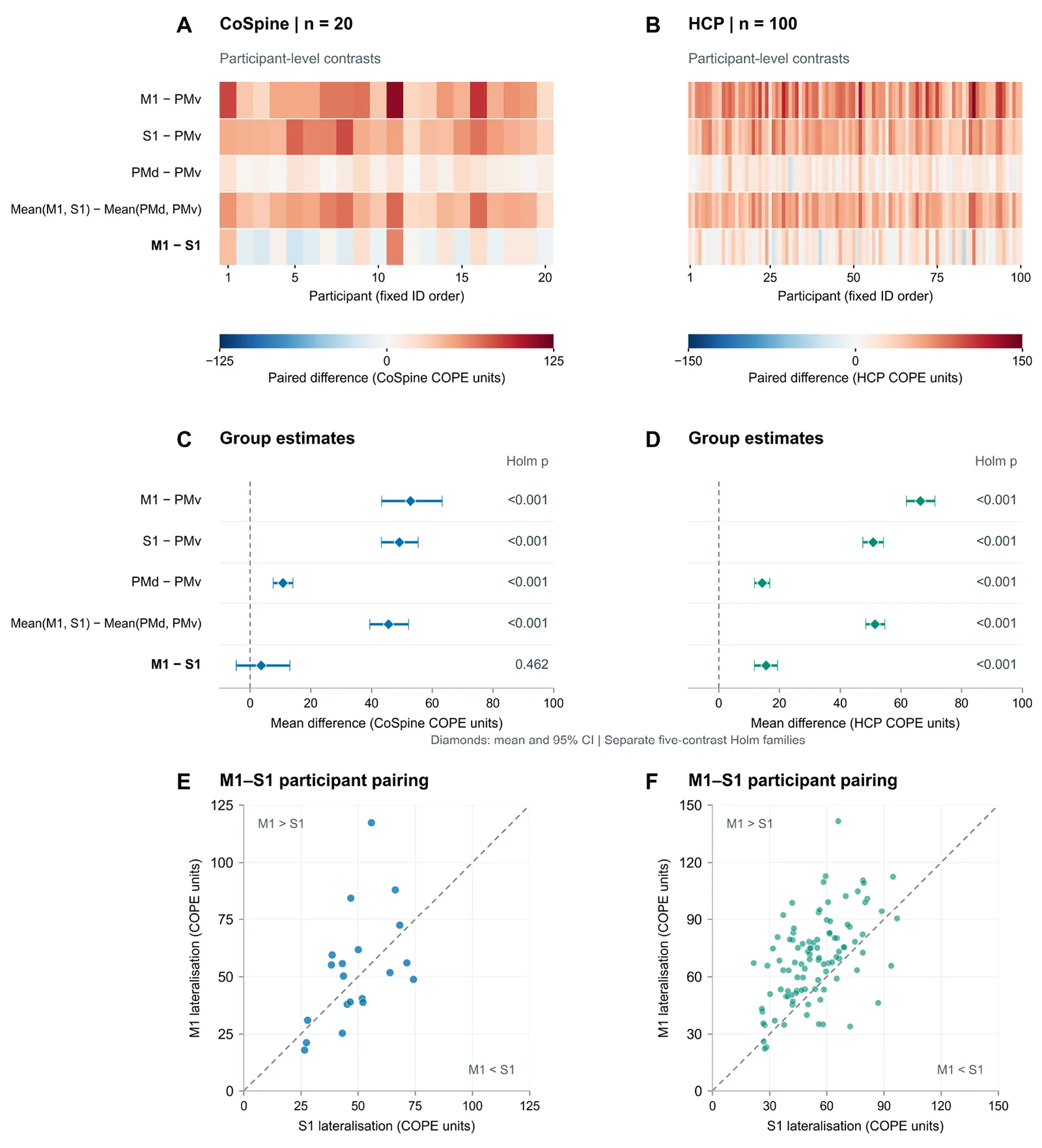
Individual patterns and group estimates of regional contrasts. (A, B) Five within-participant regional contrasts in CoSpine (n = 20) and HCP (n = 100). Heatmap columns follow fixed participant-ID order; rows follow the displayed contrast order. Colors show signed differences on separate, zero-centered cohort scales. (C, D) Mean differences and pointwise 95% percentile-bootstrap confidence intervals from 10,000 participant resamples. Displayed p-values are Holm-adjusted across five comparisons within each cohort, using exact two-sided mean sign-flip tests for CoSpine and 100,000 Monte Carlo sign flips with a plus-one correction for HCP. Dashed lines denote zero. (E, F) Participant-level M1–S1 pairing; dashed identity lines indicate equal lateralization. Mean(M1, S1) - Mean(PMd, PMv) compares equally weighted regional pairs. All estimates retain cohort-specific COPE units; these displays do not constitute a between-cohort test.

**Table 1:** Cohort and regional analysis sample

Characteristic	CoSpine	HCP
Available / analyzed participants	22 / 20	100 / 100
Analysed runs	40	200
Complete four-region cases	20	100
Age, mean (SD); range, years	52.55 (9.96); 37–74	Not available for exact sample
Recorded sex, male/female	10 / 10	Not available for exact sample
Handedness	Right-handed	Not summarised for exact sample
Task organisation	Separate left-/right-hand grasping runs	Both hands in each LR/RL run
Repetition time, s	2.68	0.72
Volumes per run	115; one run with 170	284
Atlas regions	M1, S1, PMd, PMv	Same four bilateral regions

a CoSpine excludes sub-03 and sub-04 from paired endpoints. LR/RL are HCP phase-encoding directions. Age and sex refer to the final CoSpine participants, not the original 22-person cohort. HCP demographics are not inferred from a general release description.

**Table 2:** Composite and regional lateralization

Cohort	Endpoint	n	Mean [95% CI]	*d_z_*	Raw p	Holm p
CoSpine	Composite	20	28.059 [23.718, 32.551]	2.721	< 0.001	–
CoSpine	M1	20	52.686 [43.062, 63.805]	2.174	< 0.001	< 0.001
CoSpine	S1	20	49.033 [43.057, 55.255]	3.444	< 0.001	< 0.001
CoSpine	PMd	20	10.661 [7.420, 14.403]	1.312	< 0.001	< 0.001
CoSpine	PMv	20	−0.144 [− 2.901, 2.406]	−0.023	0.917	0.917
HCP	Composite	100	35.947 [33.743, 38.185]	3.192	< 0.001	–
HCP	M1	100	69.470 [65.018, 73.904]	3.090	< 0.001	< 0.001
HCP	S1	100	53.907 [50.593, 57.308]	3.154	< 0.001	< 0.001
HCP	PMd	100	17.352 [15.461, 19.255]	1.762	< 0.001	< 0.001
HCP	PMv	100	3.061 [0.643, 5.454]	0.249	0.035	0.035

a Means and intervals are in cohort-specific COPE units. Intervals are pointwise percentile intervals from 10,000 participant bootstrap resamples. CoSpine p-values use exact two-sided mean sign flips; HCP p-values use exact binomial sign tests. These tests target different null properties.

b Composite tests are single endpoints; the four regional tests form a separate Holm family in each cohort. *d_z_* is mean divided by sample SD. A dash means no family adjustment applies. Small p values are displayed as < 0.001; numerical source tables preserve full precision.

**Table 3:** Five within-participant regional contrasts

Cohort	Contrast	n	Mean difference [95% CI]	*d_z_*	Holm p
CoSpine	M1 – PMv	20	52.830 [43.280, 63.282]	2.275	< 0.001
CoSpine	S1 – PMv	20	49.177 [43.204, 55.373]	3.413	< 0.001
CoSpine	PMd – PMv	20	10.805 [7.615, 14.117]	1.413	< 0.001
CoSpine	Mean(M1, S1) – Mean(PMd, PMv)	20	45.601 [39.413, 52.179]	3.081	< 0.001
CoSpine	M1 – S1	20	3.653 [− 4.559, 13.112]	0.174	0.462
HCP	M1 – PMv	100	66.409 [61.791, 71.183]	2.780	< 0.001
HCP	S1 – PMv	100	50.846 [47.436, 54.234]	2.955	< 0.001
HCP	PMd – PMv	100	14.291 [11.772, 16.778]	1.115	< 0.001
HCP	Mean(M1, S1) – Mean(PMd, PMv)	100	51.482 [48.408, 54.663]	3.190	< 0.001
HCP	M1 – S1	100	15.563 [11.706, 19.381]	0.787	< 0.001

a Contrasts use complete within-participant regional values. Estimates retain each cohort’s COPE units and pointwise 10,000-resample percentile intervals. All five contrasts form one Holm family per cohort.

b CoSpine uses exact two-sided mean sign flips; HCP uses 100,000 Monte Carlo sign flips with a plus-one correction. No contrast was added or dropped according to significance. Full raw and adjusted p-values are supplied in Supplementary Material 2.

## Data Availability

The CoSpine motor-task dataset is publicly available through OpenNeuro (accession ds005884; https://openneuro.org/datasets/ds005884). HCP Young Adult data are available through the Human Connectome Project repository (https://www.humanconnectome.org/study/hcp-young-adult), subject to the applicable registration and data-use terms. Group-level derived results are provided in the Supplementary Information.

## Code availability

The analysis code supporting this study is available from the corresponding authors upon reasonable request.

## References

[1] Alahmadi AAS, Pardini M, Samson RS, D’Angelo E, Friston KJ, Toosy AT, Gandini Wheeler-Kingshott CAM (2015) Differential involvement of cortical and cerebellar areas using dominant and nondominant hands: An FMRI study. Hum Brain Mapp 36:5079–5100. 10.1002/hbm.22997 PMC4737094 26415818

[2] Barch DM, Burgess GC, Harms MP, Petersen SE, Schlaggar BL, Corbetta M (2013) Function in the human connectome: task-fMRI and individual differences in behavior. NeuroImage 80:169–189. 10.1016/j.neuroimage.2013.05.033 PMC4011498 23684877

[3] Bathke AC, Friedrich S, Pauly M, Konietschke F, Staffen W, Strobl N (2018) Testing mean differences among groups: multivariate and repeated measures analysis with minimal assumptions. Multivar Behav Res 53:348–359. 10.1080/00273171.2018.1446320 PMC5935051 29565679

[4] Diedrichsen J, Wiestler T, Krakauer JW (2013) Two distinct ipsilateral cortical representations for individuated finger movements. Cereb Cortex 23:1362–1377. 10.1093/cercor/bhs120 PMC3643717 22610393

[5] Fan L, Li H, Zhuo J, Zhang Y, Wang J, Chen L (2016) The Human Brainnetome Atlas: a new brain atlas based on connectional architecture. Cereb Cortex 26:3508–3526. 10.1093/cercor/bhw157 PMC4961028 27230218

[6] Friedrich S, Konietschke F, Pauly M (2019) Resampling-based analysis of multivariate data and repeated measures designs with the R package MANOVA.RM. R J 11:380–400. 10.32614/RJ-2019-051

[7] Glasser MF, Sotiropoulos SN, Wilson JA, Coalson TS, Fischl B, Andersson JL (2013) The minimal preprocessing pipelines for the Human Connectome Project. NeuroImage 80:105–124. 10.1016/j.neuroimage.2013.04.127 PMC3720813 23668970

[8] Gordon EM, Chauvin RJ, Van AN, Rajesh A, Nielsen A, Newbold DJ (2023) A somato-cognitive action network alternates with effector regions in motor cortex. Nature 617:351–359. 10.1038/s41586-023-05964-2 PMC10172144 37076628

[9] Holm S (1979) A simple sequentially rejective multiple test procedure. Scand J Stat 6:65–70. https://www.jstor.org/stable/4615733

[10] Horenstein C, Lowe MJ, Koenig KA, Phillips MD (2009) Comparison of unilateral and bilateral complex finger tapping-related activation in premotor and primary motor cortex. Hum Brain Mapp 30:1397–1412. 10.1002/hbm.20610 PMC6871138 18537112

[11] Hoshi E, Tanji J (2006) Differential involvement of neurons in the dorsal and ventral premotor cortex during processing of visual signals for action planning. J Neurophysiol 95:3596–3616. 10.1152/jn.01126.2005 16495361

[12] Jenkinson M, Beckmann CF, Behrens TE, Woolrich MW, Smith SM (2012) FSL NeuroImage 62:782–790. 10.1016/j.neuroimage.2011.09.015 21979382

[13] Porro CA, Francescato MP, Cettolo V, Diamond ME, Baraldi P, Zuiani C (1996) Primary motor and sensory cortex activation during motor performance and motor imagery: a functional magnetic resonance imaging study. J Neurosci 16:7688–7698. 10.1523/JNEUROSCI.16-23-07688.1996 PMC6579073 8922425

[14] Power JD, Barnes KA, Snyder AZ, Schlaggar BL, Petersen SE (2012) Spurious but systematic correlations in functional connectivity MRI networks arise from subject motion. NeuroImage 59:2142–2154. 10.1016/j.neuroimage.2011.10.018 PMC3254728 22019881

[15] Tomasi D, Volkow ND (2025) Associations between fMRI signal amplitude, hemispheric asymmetry, and task performance. Commun Biol 8:1439. 10.1038/s42003-025-08843-w PMC12508031 41062767

[16] Tzourio-Mazoyer N, Labache L, Zago L, Hesling I, Mazoyer B (2021) Neural support of manual preference revealed by BOLD variations during right and left finger-tapping in a sample of 287 healthy adults balanced for handedness. Laterality 26:398–420. 10.1080/1357650X.2020.1862142 33403938

[17] Van Essen DC, Smith SM, Barch DM, Behrens TE, Yacoub E, Ugurbil K (2013) The WU-Minn Human Connectome Project: an overview. NeuroImage 80:62–79. 10.1016/j.neuroimage.2013.05.041 PMC3724347 23684880

[18] Verstynen T, Diedrichsen J, Albert N, Aparicio P, Ivry RB (2005) Ipsilateral motor cortex activity during unimanual hand movements relates to task complexity. J Neurophysiol 93:1209–1222. 10.1152/jn.00720.2004 15525809

[19] Wei Z, Lin X, Zhang L, Guo L, Liu J, Hu L, Liu Y, Kong Y (2025) CoSpine open-access simultaneous cortico-spinal fMRI database of thermal pain and motor tasks. Sci Data 12:1696. 10.1038/s41597-025-05982-x PMC12559740 41145516

[20] Winkler AM, Ridgway GR, Webster MA, Smith SM, Nichols TE (2014) Permutation inference for the general linear model. NeuroImage 92:381–397. 10.1016/j.neuroimage.2014.01.060 PMC4010955 24530839

